# Human Papillomavirus Types 52 and 58 Are Prevalent in Uterine Cervical Squamous Lesions from Japanese Women

**DOI:** 10.4061/2011/246936

**Published:** 2011-05-26

**Authors:** Kazuhiro Takehara, Tamaki Toda, Toshinao Nishimura, Junichi Sakane, Yosuke Kawakami, Tomoya Mizunoe, Morie Nishiwaki, Kiyomi Taniyama

**Affiliations:** ^1^Department of Obstetrics and Gynecology, National Hospital Organization Kure Medical Center and Chugoku Cancer Center, Kure 737-0023, Japan; ^2^Department of Diagnostic Pathology, National Hospital Organization Kure Medical Center and Chugoku Cancer Center, Kure 737-0023, Japan; ^3^GLab Pathology Center Co., Ltd., Sapporo 060-0009, Japan; ^4^Institute for Clinical Research, National Hospital Organization Kure Medical Center and Chugoku Cancer Center, Kure 737-0023, Japan

## Abstract

*Objective*. To estimate the prevalence and genotypes of high-risk human papillomavirus (HPV) focusing HPV 16, 18, 52, and 58 in Japan. *Methods*. Liquid-base cytology specimens were collected from Japanese women (*n* = 11022), aged 14–98. After classifying cytodiagnosis, specimens were analyzed for HPV DNA by the multiplex polymerase chain reaction method, where 1195 specimens were positive for cervical smear, except adenomatous lesions. *Result*. HPV genotypes were detected in 9.5% of NILM and 72.2% of ASC-US or more cervical lesions. In positive cervical smears, HPV genotypes were HPV 52 at 26.6%, HPV 16 at 25.2%, HPV 58 at 21.8%, and HPV 18 at 7.1%. Most patients infected with HPV 16 were between 20–29 years old, decreasing with age thereafter. As for HPV 52 and 58, although the detection rate was high in 30- to 39-year-olds, it also was significant in the 50s and 60s age groups. *Conclusion*. In Japan, as a cause of abnormal cervical cytology, HPV52 and 58 are detected frequently in addition to HPV 16. In older age groups, HPV 52 and 58 detection rates were higher than that observed for HPV 16. After widespread current HPV vaccination, we still must be aware of HPV 52 and 58 infections.

## 1. Introduction

Cervical cancer is the major cause of death from gynecological cancer worldwide, even though screening with cervical cytological testing (the Papanicolaou (Pap) test) has been available for over 50 years. In recent years, molecular biology has firmly established a causal relationship between persistent infection with high-risk human papillomavirus (HPV) genotypes and cervical cancer. Hence, geographic variations in HPV type distributions should be an important consideration [1]. A meta-analysis including 14 Japanese studies revealed that high-risk types considered carcinogenic or probably carcinogenic included HPV 16, 18, 31, 33, 35, 52, and 58 in Japan [2, 3]. Muñoz et al. reported that HPV 16 and 18 were associated with 73.5% of invasive cervical cancer (ICC) in Southeast Asia, 76.9% in Northern Africa, and 71.5% in Europe/North America and HPV 52 and 58 were detected in 6.1% of ICC cases in Southeast Asia, 1.5% in Northern Africa, and 1.1% in Europe/North America [4]. In Japan, however, HPV 16 and 18 were less frequently identified (58.8%) and HPV 52 and 58 were more common (13.7%) [2]. 

 Recently, we have started a new trial for liquid-based cytology (LBC) with ThinPrep, in conjunction with a novel, one-step HPV-typing method using multiplex polymerase chain reaction method (PCR) in Japan (CCLBC study) under the financial support of the National Hospital Organization of Japan. The CCLBC study has been designed to perform a multihospital analysis to further assess the effort of LBC and the prevalence of HPV of the cervix.

 In this paper, we report the prevalence of the HPV genotypes focusing on HPV 16, 18, 52, and 58, using LBC in Japan.

## 2. Materials and Methods

This study was based on a CCLBC study. That is, cervical cytology samples were obtained by a Broom Brush from 8 hospitals and institutes (Kure Medical Center/Chugoku Cancer Center, JA Hiroshima General Hospital, Hiroshima-Nishi Medical Center, Chugoku Central Hospital, Fukuyama Medical Center, Fukuyama City Hospital, Affiliated Hospitals of Clinical Laboratory for Fukuyama City Medical Association, and Hamada Medical Center). To compare LBC with the conventional Pap smear, a split-sample method was employed. Cytology was diagnosed by the most severe lesion, as identified by LBC or the conventional Pap smear. Between October 2007 and March 2010, 11022 specimens (9760 negative for intraepithelial lesion or malignancy (NILM), 1195 abnormal smear findings, except adenomatous lesions) excluding inadequate specimens were available for analysis. Written informed consent was obtained from all patients.

 Pap-stained specimens were screened by cytotechnologists and were classified according to the Bethesda system 2001. All cytotechnologists and cytopathologists participating in the study had been authorized by Cytyc Corporation for use of the ThinPrep test.

**Figure 1:**
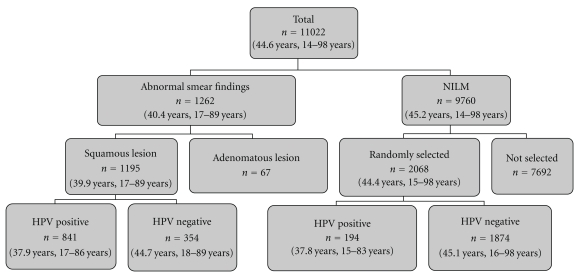
Objectives profile number in the parenthesis: average age, range.

 We randomly selected 2068 specimens from the 9760 samples of normal cytology. These 2068 normal cytology specimens and 1195 abnormal cytology specimens were used for detecting HPV genotypes (Figure 1). The residual cells in the preservative medium were used for the preparation of DNA samples. HPV-DNA testing was performed using the multiplex PCR method (PapiPlex) at the GLab Pathology Center Co., Ltd. (Sapporo, Japan). It can detect 16 high- and low-risk HPV genotypes (genotypes 6, 11, 16, 18, 30, 31, 33, 35, 39, 45, 51, 52, 56, 58, 59, and 66) in a single tube. Nishiwaki et al. provided more details about the multiplex PCR method (PapiPlex) in their paper [5].

## 3. Results

This analysis included 11022 Japanese women. The mean age of the study subjects was 44.6 years (range, 14–98 years). Of these, 9760 cases did not exhibit abnormal cytological findings (NILM). ASC-US or more were found in 1262 cervical smears. Of the total, 1195 were squamous lesions (169 ASC-US, 25 atypical squamous cells, cannot exclude high-grade squamous intraepithelial lesion (ASCH), 447 low-grade squamous intraepithelial lesion (LSIL), 449 high-grade squamous intraepithelial lesion (HSIL), and 105 squamous cell carcinoma (SCC)) and 67 were adenomatous lesions. The age and distribution were almost the same in the two groups (Table 1). There were 841 patients of abnormal cytological findings, and 194 of NILM were positive for HPV genotyping. The mean age of HPV-positive patients with abnormal squamous lesion was 37.9 years (range, 17–86 years), and that of patients with NILM was 37.8 years (range, 15–83). 

HPV prevalence was 9.4% in 2068 women with NILM, 45.6% (77/169) in ASC-US, 68.0% (17/22) in ASCH, 65.1% (291/447) in LSIL, 83.3% (374/449) in HSIL, and 78.1% (82/105) in SCC (Table 2).

 In NILM, the detected HPV genotypes and positive rates were HPV 52 (23.7%, 46/194), HPV 58 (17.0%, 33/194), HPV 16 (15.5%, 30/194), HPV 18 (10.8%, 21/194), and HPV 39 (10.3%, 20/194), in order of frequency. In abnormal smear findings, HPV genotypes and positive rates were HPV 16 (27.5%, 231/841), HPV 52 (27.3%, 230/841), HPV 58 (26.9%, 193/841), HPV 51 (9.6%, 81/841), and HPV 31 (8.6%, 72/841), in order of frequency. HPV genotypes and rates of SCC in HPV-positive cases were HPV 16 (16.9%, 44/261), HPV 18 (8.2%, 6/73), HPV 52 (7.6%, 21/276), and HPV 58 (5.8%, 13/226), in order of frequency (Table 2).

**Figure 2:**
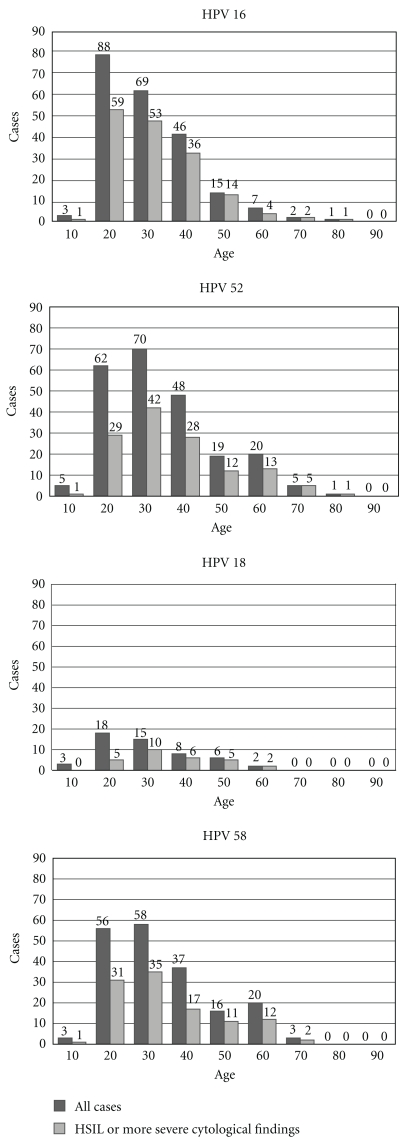
Prevalence of HPV and cytological findings by age.

 In NILM, the prevalence of HPV 16, 18, 52, and 58 was high among the 20- to 39-year olds. HPV 16, 18, 52, and 58 detection rates in HSIL or more severe cytological findings were higher than those in women with cytological abnormalities across all age groups. In HSIL or more, the prevalence of HPV 16 was highest among women aged 20 to 29 years old, decreasing with age thereafter. As for HPV 52 and HPV 58, although the detection rate was high in 30- to 39-year olds, it had increased in the 50s and 60s again (Table 3, Figure 2).

## 4. Discussion

Recent studies revealed that at least 13 HPV types, including types 16, 18, 31, 33, 35, 39, 45, 51, 52, 56, 58, 59, and 68 are commonly associated with ICC [4, 9–11]. While HPV 16 is the most prevalent type in the world, it has been reported that the frequency of other high-risk HPV types varies by region. In this study, to investigate the prevalence of HPV genotypes in Japan, especially HPV 52 and 58, cervical smear HPV-DNA testing was performed by a PCR method. For adenomatous lesions, its status as a precancer lesion has been uncertain, so we excluded it from this consideration. HPV was detected in 9.4% of patients with NILM and in 70.4% of patients with cytological abnormalities. In a meta-analysis, the overall HPV prevalence in Japanese women with normal cytology was only 10.2% [2]. Some previous studies have shown the overall HPV prevalence in Japanese women with normal cytology to be 9.7–22.5% (Table 4) [3, 6–8]. In the present study, the mean age of normal cytology cases was 44.4 years and the mean age of HPV-positive cases was 37.8 years. Because HPV infection was most frequently detected in young women aged 15–25 years, HPV detection rate was considered to be high.

 In abnormal smear cases, HPV genotypes and positivity were HPV 16 at 27.5%, 52 at 27.3%, 58 at 26.9%, 51 at 9.6%, and 31 at 8.6%, in order of frequency. These data indicate that these HPV subtypes mainly involve abnormal cervical lesions. Miura et al. performed a meta-analysis of HPV type prevalence and type-specific risks for cervical carcinogenesis in Japan. They reported that 21 kinds of HPV types were detected from invasive cervical cancer, where the HPV genotypes and positive rates were HPV 16, 18, 52, 58, 33, 31, 35, and 51, in order of frequency [2]. Comparing data with the pooled analysis of Muñoz et al., they pointed out that HPV 16 and 18 were less frequent and HPV 52 and 58 were more common in Japan. In this study, rates of SCC in HPV-positive cases were HPV 16 at 16.9%, 18 at 8.2%, 52 at 7.6%, and 58 at 5.8%, in order of frequency. These data indicate that HPV 16 and 18 are the major types in all continents and HPV 52 and 58 are the commonest types in Japan. Recently, Onuki et al. also reported that HPV 16 and 18 were less frequently identified in ICC cases in Japan compared with Southeast Asia, North America, and Europe, with HPV 31, 33, 52, and 58 accounting for approximately 20% of ICC [3]. For such reasons, a different strategy for HPV vaccination will be necessary in Japan, although HPV 16 and 18 vaccines have been licensed since 2009. Onuki et al. suggested that HPV 16 and 18 vaccines may provide 65% protection against ICC in Japan, which is lower than the initial proposal that current HPV vaccines which are directed against only HPV 16 and l8 are considered to prevent a majority (>70%) of cervical cancer world-wide [12, 13]. The lower rate of effectiveness arises from the fact that HPV 16 and 18 are less frequently identified and HPV 31, 33, 52, and 58 are more common in ICC cases in Japan compared to Southeast Asia, Northern Africa, Europe, and North America. 

 Recently, the interpretation of geographical variations of HPV type distribution has been requiring careful consideration in view of the differences in sensitivity of the HPV detection assays [1]. For example, it has been reported that the sensitivity of MY09/11-based assays was greater for HPV 52 than GP5+/6+. There were no differences in DNA detection for HPV 16, 18, 33, and 45 across different laboratories and assays [14]. Conversely, HPV 31, 35, 52, and 6 did show assay differences in sensitivity and specificity that should be considered when interpreting results from different laboratories [15]. Taniyama et al. pointed out the same issue. They reported that the prevalence of HPV 52 infection may have been underestimated in previous studies and the HPV 52 type could be more prevalent than what has been reported in Asia, and as a result, the vaccination against HPV 16 and 18 would be less effective in Asia than that expected in the West [16].

 Clinical studies of  HPV vaccines have demonstrated close to 100% protection against HPV 16- and HPV 18-related infections and diseases [17–19], implying potential cross-protection against HPV 31, 33,  45, 52, and 58 [18, 19]. Paavonen et al. assessed the HPV 16 and 18 vaccine efficacy for young women aged 15–25 years. They reported that vaccine efficacy against CIN2 or more (CIN2+) with a composite endpoint of the most prevalent nonvaccine oncogenic HPV types (i.e., 31, 33, 45, 52, and 58) in invasive cervical cancer was greater than 50%. Tangible data of vaccine efficacy for CIN2+ against HPV 31,33, 45, 52, and 58 was 92.0%, 51.9%, 100%, 14.3%, and 64.5% respectively. Although significant vaccine efficacy against HPV 31, 33, and 45 was noted for 6-month persistent infections, no significant vaccine efficacy against HPV 52 and 58 was observed for 6-month persistent infections or 12-month persistent infections and CIN2+. This result supports the robustness of cross-protection against HPV types 31, 33, and 45, and vaccine efficacy against HPV 52 and 58 has a certain level of effect [20].

 We also need to focus on HPV prevalence by age. HPV infection was most frequently detected in young women aged 15–25 years, and a second peak was observed in women aged 55 years or older, which is also consistent with results from African, American, and European populations [21], although the reason for the second peak is unknown [3]. Major risk factors for cervical cancer are high-risk HPV infection and persistent infection. One of the persistent risk factors is old age. In our analysis, older women have a high detection rate of HPV 52 and 58. Lindau et al., who estimated the prevalence and genotypes of high-risk HPV among women aged 57–85, also reported that the prevalence of HPV 52 (12.9%) and 58 (12.5%) was higher than that of HPV 16 (9.7%) [22]. 

 In the future, HPV typing in conjunction with cervical cytology testing could be used for uterine cancer screening or in a follow-up program after conservative treatment of uterine cervical lesions since around 10% of NILM patients were HPV-positive. The natural history of HPV 52 and 58 is still unknown. Even after current widespread HPV vaccination, cross-protection against HPV 52 and 58 after the age of 30 remains incompletely understood, so attention should be paid to geographical distribution and causal significance of HPV types for uterine cervical lesions. A more sensitive and accurate method for HPV typing with corresponding clinical research will permit better understanding of the effects of the HPV genotype.

## 5. Conclusions

In Japan, as a cause of abnormal cervical cytology, HPV 52 and HPV 58 cannot be ignored. Even after current widespread HPV vaccination, smear abnormalities focusing on HPV 52 and HPV 58 infection should be carefully followed up.

## Figures and Tables

**Table 1:** Characteristics at registration of all patients.

Characteristic	*n* = 11022	*n* = 2068*
Age		
Mean (range)	44.6 y (14–98 y)	45.2 y (15–98 y)
10–19 years	123 (1.1%)	22 (1.1%)
20–29 years	1657 (15.0%)	331 (16.2%)
30–39 years	2658 (24.1%)	461 (22.5%)
40–49 years	2703 (24.5%)	537 (26.2%)
50–59 years	2108 (19.1%)	403 (19.7%)
60–69 years	1117 (10.1%)	197 (9.6%)
70–79 years	517 (4.7%)	91 (4.4%)
80–89 years	132 (1.2%)	23 (1.1%)
90–99 years	7 (0.1%)	3 (0.14%)

Symptoms		
None	9859	1869
Bleeding	746	127
Others	414	71
Unknown	3	1

Cytodiagnosis (The Bethesda System 2001)		
NILM	9760*	
Abnormal smear	1262	
ASCUS	169	
ASCH	25	
LSIL	447	
HSIL	449	
SCC	105	
Adenomatous lesions	67	

An abnormal smear is defined as one with atypical squamous cells of undetermined significance (ASCUS) or more severe cytological findings.*2068 from 9760 NILM patients randomly assigned to detect HPV genotype.

**Table 2:** HPV prevalence.

	No. of cases		HPV type															
	Multiplex PCR	Positive for HPV	6	11	16	18	30	31	33	35	39	45	51	52	56	58	59	66
NILM*	2068	194	11 (5)	3 (1)	30 (12)	21 (7)	5 (2)	11 (3)	8 (4)	3	20 (7)	1	15 (8)	46 (19)	18 (3)	33 (13)	4 (1)	15 (5)
ASC-US	169	77	4 (1)	—	5 (1)	4 (1)	1	4 (2)	5 (2)	6 (1)	5 (1)	1	10 (1)	21 (2)	—	17 (7)	—	5 (1)
ASC-H	25	17	—	—	5 (2)	—	—	3	2 (2)	1	2 (2)	—	1 (1)	4 (2)	—	3 (1)	2 (1)	—
LSIL	447	291	19 (12)	—	51 (28)	20 (13)	3 (1)	20 (13)	12 (6)	7 (5)	27 (15)	7 (4)	31 (15)	74 (38)	45 (14)	64 (35)	11 (8)	31 (17)
HSIL	449	374	5 (5)	1 (1)	126 (59)	22 (12)	5 (4)	38 (18)	18 (10)	6 (3)	24 (14)	5 (5)	34 (20)	110 (53)	16 (8)	96 (47)	5 (4)	19 (15)
SCC	105	82	2 (2)	—	44 (14)	6 (3)	1 (1)	7 (3)	7 (4)	1	1	—	5 (5)	21 (11)	1 (1)	13 (7)	—	3 (1)

Total	3263	1035	41 (25)	4 (2)	261 (116)	73 (36)	15 (8)	83 (39)	52 (28)	24 (9)	79 (39)	14 (9)	96 (50)	276 (125)	80 (26)	226 (110)	22 (14)	73 (39)

Number in the parenthesis: number of cases with multiple HPV infections.

*Patients who were randomly selected from 9760 NILM patients.

**Table:** (a) Women without cytological abnormalities (NILM)

HPV type	10–19 *n* = 22	20–29 *n* = 331	30–39 *n* = 461	40–49 *n* = 537	50–59 *n* = 403	60–69 *n* = 197	>70 *n* = 117	All *n* = 2068
HPV 16	1 (4.5%)	7 (2.1%)	12 (2.6%)	8 (1.5%)	1 (0.2%)	0	1 (0.9%)	30 (1.5%)
HPV 18	1 (4.5%)	8 (2.4%)	6 (1.3%)	4 (0.7%)	1 (0.2%)	1 (0.5%)	0	21 (1.0%)
HPV 52	1 (4.5%)	15 (4.5%)	7 (1.5%)	8 (1.5%)	5 (1.3%)	7 (3.6%)	3 (2.6%)	46 (2.2%)
HPV 58	1 (4.5%)	10 (3.0%)	15 (3.3%)	4 (0.7%)	0	2 (1.0%)	1 (0.9%)	33 (1.6%)

**Table:** (b) Women with cytological abnormalities

HPV type	10–19 *n* = 21	20–29 *n* = 271	30–39 *n* = 358	40–49 *n* = 290	50–59 *n* = 153	60–69 *n* = 69	>70 *n* = 33	All *n* = 1195
HPV 16	3 (14.0%)	88 (32.5%)	69 (19.3%)	46 (15.9%)	15 (9.8%)	7 (10.1%)	3 (9.1%)	231 (19.3%)
HPV 18	3 (14.0%)	18 (6.6%)	15 (4.2%)	8 (2.8%)	6 (3.9%)	2 (2.9%)	0	52 (4.4%)
HPV 52	5 (23.8%)	62 (22.9%)	70 (19.6%)	48 (16.6%)	19 (12.4%)	20 (29.0%)	6 (18.2%)	230 (19.2%)
HPV 58	3 (14.0%)	56 (20.7%)	58 (16.2%)	37 (12.8%)	16 (10.5%)	20 (29.0%)	3 (9.1%)	193 (16.2%)

**Table:** (c) Women with cytological abnormalities of HSIL or more

HPV type	10–19 *n* = 4	20–29 *n* = 127	30–39 *n* = 183	40–49 *n* = 117	50–59 *n* = 64	60–69 *n* = 41	>70 *n* = 18	All *n* = 554
HPV 16	1 (25.0%)	59 (46.5%)	53 (29.0%)	36 (30.8%)	14 (21.9%)	4 (9.8%)	3 (16.7%)	170 (30.7%)
HPV 18	0	5 (3.9%)	10 (5.5%)	6 (5.1%)	5 (7.8%)	2 (4.9%)	0	28 (5.1%)
HPV 52	1 (25.0%)	29 (22.8%)	42 (23.0%)	28 (23.9%)	12 (18.8%)	13 (31.7%)	6 (33.3%)	131 (23.6%)
HPV 58	1 (25.0%)	31 (24.4%)	35 (19.1%)	17 (14.5%)	11 (17.2%)	12 (29.3%)	2 (11.1%)	109 (19.7%)

**Table 4:** Reports of HPV prevalence in cytologically normal women.

Author	Year	Number of patients	Duration of sample collection	Mean age (range)	HPV prevalence
Yoshikawa et al. [6]	1999	130	1995–1996	40.7 (unknown)	14.6% (19/130)
Sasagawa et al. [7]	2001	1562	1995–1999	unknown (16–72)	9.7% (151/1562)
Asato et al. [8]	2004	3249	1993–1995	52.4 (18–85)	10.2% (333/3249)
Onuki et al. [3]	2009	1517	1999–2007	35.0 (15–78)	22.5% (342/1517)
Present report		2068	2007–2009	44.4 (15–98)	9.4% (194/2068)
